# Timing of risk reducing mastectomy in breast cancer patients carrying a *BRCA1/2* mutation: retrospective data from the Dutch HEBON study

**DOI:** 10.1007/s10689-015-9788-x

**Published:** 2015-02-21

**Authors:** M. R. Wevers, M. K. Schmidt, E. G. Engelhardt, S. Verhoef, M. J. Hooning, M. Kriege, C. Seynaeve, M. Collée, C. J. van Asperen, R. A. E. M. Tollenaar, L. B. Koppert, A. J. Witkamp, E. J. T. Rutgers, N. K. Aaronson, M. A. Rookus, M. G. E. M. Ausems

**Affiliations:** 1 Division of Psychosocial Research and Epidemiology, The Netherlands Cancer Institute, PO Box 90203, 1006 BE Amsterdam, The Netherlands; 2 Division of Biomedical Genetics, University Medical Center Utrecht, KC.04.084.2, PO Box 85090, 3508 AB Utrecht, The Netherlands; 3 Division of Molecular Pathology, The Netherlands Cancer Institute, PO Box 90203, 1006 BE Amsterdam, The Netherlands; 4 Family Cancer Clinic, The Netherlands Cancer Institute, PO Box 90203, 1006 BE Amsterdam, The Netherlands; 5 Family Cancer Clinic, Department of Medical Oncology, Erasmus MC Cancer Institute, PO Box 5201, 3008 AE Rotterdam, The Netherlands; 6 Department of Clinical Genetics, Erasmus University Medical Center, PO Box 2040, 3000 CA Rotterdam, The Netherlands; 7 Department of Clinical Genetics, Leiden University Medical Center, PO Box 9600, 2300 RC Leiden, The Netherlands; 8 Division of Surgery, Leiden University Medical Center, PO Box 9600, 2300 RC Leiden, The Netherlands; 9 Division of Surgery, Erasmus MC Cancer Institute, PO Box 5201, 3008 EA Rotterdam, The Netherlands; 10 Division of Surgery, University Medical Center Utrecht, PO Box 85500, 3508 GA Utrecht, The Netherlands; 11 Division of Surgery, The Netherlands Cancer Institute, PO Box 90203, 1006 BE Amsterdam, The Netherlands

**Keywords:** *BRCA1*, *BRCA2*, Breast neoplasms, Risk reducing mastectomy

## Abstract

It is expected that rapid genetic counseling and testing (RGCT) will lead to increasing numbers of breast cancer (BC) patients knowing their *BRCA1/2* carrier status before primary surgery. Considering the potential impact of knowing one’s status on uptake and timing of risk-reducing contralateral mastectomy (RRCM), we aimed to evaluate trends over time in RRCM, and differences between carriers identified either before (predictively) or after (diagnostically) diagnosis. We collected data from female *BRCA1/2* mutation carriers diagnosed with BC between 1995 and 2009 from four Dutch university hospitals. We compared the timing of genetic testing and RRCM in relation to diagnosis in 1995–2000 versus 2001–2009 for all patients, and predictively and diagnostically tested patients separately. Of 287 patients, 219 (76 %) had a diagnostic *BRCA1*/2 test. In this cohort, the median time from diagnosis to DNA testing decreased from 28 months for those diagnosed between 1995 and 2000 to 14 months for those diagnosed between 2001 and 2009 (*p* < 0.001). Similarly, over time women in this cohort underwent RRCM sooner after diagnosis (median of 77 vs. 27 months, *p* = 0.05). Predictively tested women who subsequently developed BC underwent an *immediate* RRCM significantly more often than women who had a diagnostic test (21/61, 34 %, vs. 13/170, 7.6 %, *p* < 0.001). Knowledge of carrying a *BRCA1/2* mutation when diagnosed with BC influenced decisions concerning primary surgery. Additionally, in more recent years, women who had not undergone predictive testing were more likely to undergo diagnostic DNA testing and RRCM sooner after diagnosis. This suggests the need for RGCT to guide treatment decisions.

**Electronic supplementary material:**

The online version of this article (doi:10.1007/s10689-015-9788-x) contains supplementary material, which is available to authorized users.

## Introduction

Female *BRCA1* or *BRCA2* gene mutation carriers have an increased risk of developing breast cancer of 27–88 %, and a maximum lifetime risk of developing ovarian cancer of 6–59 % [1, 2]. Once diagnosed with unilateral breast cancer, *BRCA1/2* mutation carriers have a 16–55 % risk of developing contralateral breast cancer within 25 years, depending on, among other factors, age at first diagnosis and the mutated gene [3, 4].

In the case of a favorable disease stage and prognosis, mutation carriers diagnosed with breast cancer may opt for a risk reducing contralateral mastectomy (RRCM), which reduces the risk of contralateral breast cancer by more than 90 %, with increasing evidence of improved breast cancer specific survival [5–9]. The reported uptake of RRCM in such women ranges from 18 to 65 % [10–13]. The period of follow-up in these studies varied between a few months to several years after genetic test disclosure or breast cancer diagnosis.

Genetic testing of the *BRCA1/2* genes became available from 1994 onwards. In current practice, breast cancer patients at risk of having hereditary cancer are typically referred for genetic counseling and diagnostic DNA testing *after* their primary treatment [14–17]. In such cases, affected carriers may consider undergoing a delayed RRCM [18]. Unaffected women who become aware of their carrier status via a predictive DNA test (i.e. while still asymptomatic) and subsequently develop breast cancer, may consider an immediate RRCM (i.e. at the time of the therapeutic surgery).

However, genetic counseling and testing (GCT) can also be offered to high-risk women between breast cancer diagnosis and primary surgical treatment (*rapid genetic counseling and testing,* or *RGCT*). Being aware of one’s carrier status may influence treatment decisions, including type of surgery, use of adjuvant radiotherapy and whether to undergo an immediate RRCM [19–21]. Such knowledge may be particularly important for high risk women who are determined not to carry a *BRCA1/2* mutation, as their risk of developing contralateral breast cancer may be substantially lower than initially suspected [4, 22, 23].

It is expected that RGCT will become widely available in the near future. Information on uptake and timing of prophylactic measures chosen by carriers diagnosed with breast cancer with a favorable prognosis is therefore increasingly relevant. Such information can help breast cancer specialists to better understand the place of genetic testing in the clinical pathway of breast cancer diagnosis and treatment, and ultimately can contribute to optimal multidisciplinary treatment and care of women with breast cancer [13, 22, 24].

The objective of the current study was to describe trends over the years 1995–2009 in the timing of genetic testing and of prophylactic mastectomy in breast cancer patients carrying a *BRCA1*/2 mutation. More specifically, our aims were to describe (1) the timing of genetic testing in relation to breast cancer diagnosis; (2) the uptake of immediate RRCM (i.e., at the time of breast cancer surgical therapy) and of delayed RRCM; and (3) the timing of RRCM in relation to diagnosis.

## Materials and methods

### Study population

The Dutch HEBON study (Hereditary Breast and Ovarian cancer study, the Netherlands) is a retrospective cohort study of members of families with a *BRCA1* or *BRCA2* mutation from 1994 onwards, with a prospective follow-up [25]. The HEBON study was approved by the medical ethical committees of all centers that recruited patients, and all individuals provided informed consent. The database includes information on DNA test results, occurrence of cancer, treatment, and risk-reducing surgery. *BRCA1/2* mutation testing could be performed before (i.e., predictive) or after breast cancer diagnosis (i.e., diagnostic) (supplementary Figure 1). Within the HEBON-database, data on tumor characteristics and treatment were available for a subset of 329 female *BRCA1/2* mutation carriers who were diagnosed with breast cancer (both in situ and invasive) from 1995 onwards and were treated in the Erasmus University Medical Center (Erasmus MC) Cancer Institute, the Netherlands Cancer Institute (NKI), the University Medical Center Utrecht (UMCU) or the Leiden University Medical Center (LUMC), and were counseled in the departments of clinical genetics in those hospitals. Of those 329 carriers, 287 women had no distant metastases and/or other type(s) of cancer at time of breast cancer diagnosis, were a proven *BRCA1/2* mutation carrier, had date of DNA test result available, and had type of breast cancer surgery available, and could therefore be included in the study.

### Exclusion criteria for choice of RRCM

For questions concerning choice of *immediate* RRCM, exclusion criteria were: (e) synchronous bilateral breast cancer; and (f) breast cancer after a prophylactic bilateral mastectomy; together n = 56. For questions concerning choice of *delayed* RRCM, additional exclusion criteria were: (g) diagnosis of (metastases of) other type(s) of cancer at time of DNA test result; (h) having received treatment and counseling in the LUMC due to too many missing data on prophylactic mastectomy; (i) missing data on RRCM; and (j) having had a RRCM before contralateral breast cancer diagnosis, i.e., being diagnosed with breast cancer despite having undergone a RRCM; together n = 80. A contralateral mastectomy without information whether it was prophylactic or therapeutic was considered a RRCM (n = 1).

An immediate RRCM is defined as removal of the contralateral breast at the same date as the therapeutic mastectomy. Delayed RRCM is defined as removal of the contralateral breast at some time after the primary surgery. In case of a prior breast conserving surgery, the ipsilateral breast is removed as well.

### Data collection

For the current study, the following data were retrieved from the HEBON-database: date of birth; tumor characteristics [ductal carcinoma in situ (DCIS) or invasive breast cancer, TNM stage, unilateral or bilateral breast cancer, date of diagnosis]; history of other cancers; mutated gene (*BRCA1* or *BRCA2*); date of DNA test result; type of surgical treatment for breast cancer; date(s) of breast cancer surgery/surgeries; type(s) and date(s) of prophylactic breast surgery/surgeries; and hospital where treatment and DNA-testing were performed. Tumor stage was categorized according to the TNM classification as included in the breast cancer guideline 2.0 (2012) of the Comprehensive Cancer Center the Netherlands [16].

### Statistical analyses

Chi square tests were used to compare choice of immediate and delayed RRCM for women who underwent a predictive DNA test versus those who had a diagnostic test, for women who were treated in 1995–2000 versus 2001–2009 separately. We chose to analyze these two time periods because, from 2001 onwards, in the Netherlands increased awareness among breast cancer specialists and probably also patients had led to larger numbers of breast cancer patients being referred for genetic counseling and testing, and GCT had become more conventional. With this division, the number of patients in both time periods still is relatively well distributed. Mann–Whitney U tests were used to compare time between breast cancer diagnosis and DNA testing for the time periods 1995–2000 versus 2001–2009. Kaplan–Meier analyses were used to compare time between first breast cancer diagnosis and risk reducing surgery, and between DNA test result and delayed risk reducing surgery for the time periods 1995–2000 versus 2001–2009. End of time under follow-up was defined as either RRCM, contralateral breast cancer diagnosis, or end of follow-up, whatever came first. Patients who had a predictive test and those who had a diagnostic test were analyzed separately, when applicable.

In addition, Cox regression was used to compare the time between diagnosis and RRCM for both time periods (categorical 1995–2000 vs. 2001–2009) adjusted for age at diagnosis (continuous variable in years), predictive versus diagnostic testing (categorical variable), nodal status (categorical positive vs. negative), and use of chemotherapy (categorical yes vs. no). Patients who had a predictive test and those who had a diagnostic test were also analyzed separately.

## Results

### Clinical and sociodemographic characteristics of the sample

From the HEBON subset of 329 women who were diagnosed with breast cancer between 1995 and 2009 in one of the participating hospitals, we included 287 *BRCA1/2* mutation carriers. The majority of the patients (77 %) had a *BRCA1* mutation (Table 1).

**Table 1:** Clinical characteristics of breast cancer patients carrying a *BRCA1/2* mutation

Characteristics	Breast cancer diagnosis 1995–2000 (N = 183)			Breast cancer diagnosis 2001–2009 (N = 104)			Total (N = 287)	*p * (1995–2000 vs. 2000–2009)
	DNA test before diagnosis	DNA test after diagnosis	*p*	DNA test before diagnosis	DNA test after diagnosis	*p*		
Number (%)	22 (12 %)	161 (88 %)		46 (44 %)	58 (56 %)			<0.0001
Affected gene, n (%)								0.01
*BRCA1*	22 (100 %)	127 (78.9 %)	0.02	37 (80.4 %)	34 (58.6 %)	0.02	220 (77 %)	
*BRCA2*	0 (0 %)	34 (21.1 %)		9 (19.6 %)	24 (41.4 %)		67 (23 %)	
Age at DNA test, years			0.42			0.23		0.40
Mean (SD)	42.9 (8.7)	44.7 (9.8)		42.4 (11.2)	44.4 (8.3)		44.1 (9.7)	
Range	23–59	20–72		22–75	24–63		20–75	
Age at breast cancer diagnosis in years			0.33			0.14		0.04
Mean (SD)	43.8 (8.8)	41.7 (9.2)		46.5 (12.0)	42.8 (8.0)		42.9 (9.5)	
Range	23–61	19–71		27–77	24–60		19–77	
DCIS or invasive breast cancer, n (%)			0.01			0.73		0.58
DCIS	4 (18.2 %)	7 (4.3 %)		4 (8.7 %)	4 (6.9 %)		19 (7 %)	
Invasive breast cancer	18 (81.8 %)	154 (95.7 %)		42 (91.3 %)	54 (93.1 %)		268 (93 %)	
Lymph node status, n (%)			0.37			0.08		0.14
Positive	6 (30 %)	60 (40.5 %)		9 (20.9 %)	20 (37.7 %)		95 (36 %)	
Negative	14 (70 %)	88 (59.5 %)		34 (79.1 %)	33 (62.3 %)		169 (64 %)	
Missing							23	
Tumor stage (n, % without unknowns) Total			0.40			0.03		0.71
Stage 0/DCIS	4 (20.0 %)	7 (6.0 %)		4 (12.9 %)	4 (10.3 %)		19 (9.2 %)	
Stage 1	9 (45.0 %)	42 (35.9 %)		15 (48.4 %)	7 (17.9 %)		73 (35.3 %)	
Stage 2	6 (30.0 %)	57 (48.7 %)		9 (29.0 %)	23 (59.0 %)		95 (45.9 %)	
Stage 3	1 (5.0 %)	11 (9.4 %)		3 (9.7 %)	5 (12.8 %)		20 (9.7 %)	
Unknown	2	44		15	19		80	

One hundred eighty-three *BRCA1/2* mutation carriers (64 %) were diagnosed with breast cancer between 1995 and 2000, and 104 (36 %) between 2001 and 2009. Mean age at breast cancer diagnosis was 42.0 years for patients diagnosed between 1995 and 2000, compared to 44.4 years for those diagnosed between 2001 and 2009 (*p* = 0.04). Other sample characteristics are described in Table 1. Of note, exclusion of DCIS cases did not alter significantly any of the findings presented in this paper (data not shown).

#### Timing of genetic counseling and testing (GCT)

Of all patients, 219 (76 %) had a diagnostic DNA test and 68 (24 %) had a predictive DNA test. Of those who had a diagnostic test, 4 received their DNA test results before primary surgery (i.e., had RGCT). Predictive DNA testing increased from 12 % for those diagnosed between 1995 and 2000 to 44 % for those diagnosed between 2001 and 2009 (*p* < 0.001) (Table 1). For patients who had a diagnostic test, the median time between breast cancer diagnosis and DNA test result decreased from 28 months [range 0–143; mean (SD) 35.7 (31.2)] for patients diagnosed between 1995 and 2000 to 14 months [range 0–80, mean (SD) 18.8 (16.5)] for those diagnosed between 2001 and 2009 (*p* < 0.001).

#### Uptake of risk reducing mastectomy

##### Immediate risk reducing contralateral mastectomy at the time of breast cancer surgery

Of the 231 patients for whom these data were available, 34 (14.7 %) had an immediate RRCM (Table 2 and supplementary flowchart). Considering the complete time period 1995–2009, breast cancer patients who had a predictive test opted for an immediate RRCM significantly more often than patients who had a diagnostic test (34.4 vs. 7.6 %, *p* < 0.001); this difference was seen in the time period 1995–2000 as well as in 2001–2009 (Table 2). Within the subgroup of predictively tested patients who underwent an immediate RRCM, there was no significant difference (*p* = 0.23) observed in choice of immediate RRCM between 1995 and 2000 (9/20, 45.0 %) versus 2001–2009 (12/41, 29.3 %).

**Table 2:** Immediate and delayed risk reducing contralateral mastectomy (RRCM), differences between patients who had a predictive test versus diagnostic test

RRCM	BREAST cancer diagnosis 1995–2000				BREAST cancer diagnosis 2001–2009				Total 1995–2009
N (%)	DNA test before diagnosis	DNA test after diagnosis	Sub-total	*p*	DNA test before diagnosis	DNA test after diagnosis	Sub-total	*p*	
Immediate RRCM	9/20 (45.0 %)	10/122 (8.2 %)	19/142 (13.4 %)	<0.0001	12/41 (29.3 %)	3/48 (6.2 %)	15/89 (16.9 %)	0.004	34/231 (14.7 %)
Delayed RRCM^a^	7/11 (63.6 %)	44/95 (46.3 %)	51/106 (48.1 %)	0.28	9/21 (42.9 %)	13/24 (54.2 %)	22/45 (48.9 %)	0.45	73/151 (48.3 %)

Only for patients with a primary unilateral breast cancer

Differences in uptake of RRCM between predictively and diagnostically tested women within and between 1995–2000 and 2001–2009 were analysed using Chi square tests

*RRCM* risk reducing contralateral mastectomy

^a^For delayed RRCM, patients from LUMC were excluded

##### Delayed risk reducing contralateral mastectomy after completion of primary breast cancer therapy

Of the 151 patients from the NKI, UMCU and Erasmus MC for whom relevant data were available, 73 (48.3 %) had a delayed RRCM after breast cancer diagnosis (Table 2). The uptake of a delayed RRCM was not significantly different between patients who had a predictive DNA test and those who had a diagnostic DNA test in neither of the time periods (Table 2). However, within the subgroup of patients who had a diagnostic test, patients who received DNA test results within a year after BC diagnosis opted for a delayed RRCM significantly more often than patients who received DNA test results more than a year after BC diagnosis [26/37 (70.3 %) versus 31/82 (37.8 %), *p* = 0.001]. This difference was also seen in the separate time periods [16/24 (66.7 %) versus 28/71 (39.4 %), *p* = 0.02 in 1995–2000, and 10/13 (76.9 %) versus 3/11 (27.3 %), *p* = 0.02 in 2001–2009].

#### Timing of risk reducing contralateral mastectomy

**Fig. 1:**
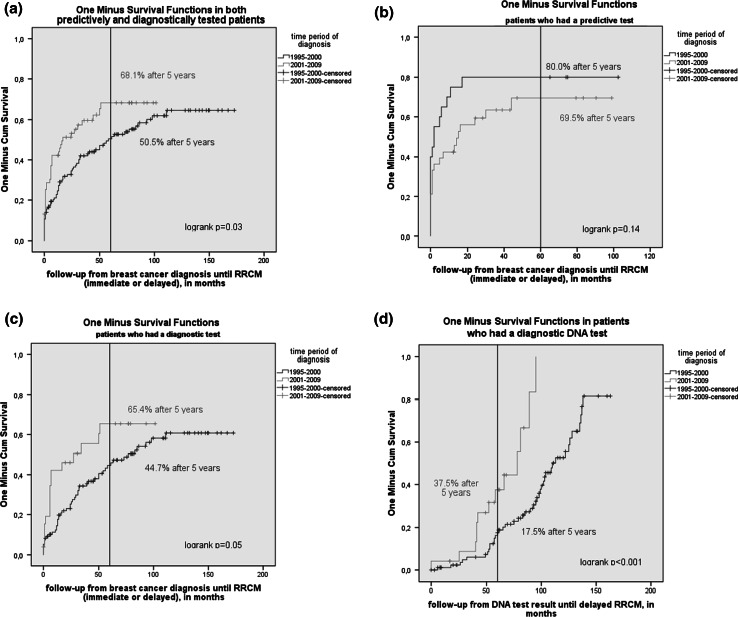
Kaplan Meier curves of cumulative RRCM incidence **a** Cumulative RRCM (immediate or delayed) incidence from time of breast cancer diagnosis comparing two time periods in both predictively and diagnostically tested patients **b** Cumulative RRCM (immediate or delayed) incidence from time of breast cancer diagnosis comparing two time periods in patients who had a *predictive* test **c** Cumulative RRCM (immediate or delayed) incidence from time of breast cancer diagnosis comparing two time periods in patients who had a *diagnostic* test **d** Cumulative *delayed* RRCM incidence from time of DNA test result in patients who had a *diagnostic* test. *RRCM risk reducing contralateral mastectomy

Overall, 50.5 % of patients who were diagnosed between 1995 and 2000 had an immediate or delayed RRCM within 5 years since breast cancer diagnosis, compared with 68.1 % for patients diagnosed between 2001 and 2009 (Fig. 1a). However, there was an indication for a difference between predictively and diagnostically tested patients, with a decrease of 80.0–69.5 % in patients who had a predictive test (Fig. 1b), and an increase of 44.7–65.4 % in patients who had a diagnostic test (Fig. 1c). In the Cox analyses, adjusted for age at diagnosis, nodal status and chemotherapy, of those diagnosed in 2001–2009, predictively tested patients were less likely, and diagnostically tested patients were more likely, to have had a RRCM than those diagnosed in 1995–2000, although both differences were not significant (Table 3). In the same multivariate model, older patients who had a predictive test were less likely to undergo a RRCM than younger patients who had a predictive test (HR 0.95, 95 % CI 0.92–0.99, *p* = 0.01).

**Table 3:** Timing of risk reducing contralateral mastectomy (RRCM) in women with *BRCA*-associated breast cancer

RRCM after breast cancer diagnosis	Breast cancer patients who had a DNA test before diagnosis (predictive) (n = 53)			Breast cancer patients who had a DNA test after diagnosis (diagnostic) (n = 127)		
	Breast cancer diagnosis 1995–2000	Breast cancer diagnosis 2001–2009	*p*	Breast cancer diagnosis 1995–2000	Breast cancer diagnosis 2001–2009	*p*
Age at RRCM in years			0.62			0.55
Mean (SD)	42.1 (7.9)	44.2 (10.0)		41.8 (7.8)	40.3 (7.7)	
Median (range)	41.5 (25–53)	45.0 (27–71)		41.0 (22–62)	40.5 (25–54)	
Median time in months between diagnosis and RRCM^a^	2	15	0.14	77	27	0.05
RRCM 2001–2009 versus 1995–2000 HR (95 % CI)^b^	0.73 (0.36–1.48)			1.72 (0.91–3.27)		

Only for patients with information on whether having undergone RRCM (from Erasmus MC Cancer Institute, NKI and UMCU), who had unilateral breast cancer. Immediate and delayed RRCM were analyzed together

*RRCM* risk reducing contralateral mastectomy

^a^Kaplan–Meier analyses for predictive and diagnostic patients

^b^Separate Cox regression models for predictive and diagnostic patients, adjusted for age at diagnosis, nodal status, and chemotherapy

In the Cox analyses for predictively and diagnostically tested patients together, adjusted for age at diagnosis, time period of diagnosis, nodal status and chemotherapy, patients who had a predictive test were more likely to have had a RRCM than patients who had a diagnostic test, with a hazard ratio of 4.49 (95 % CI 2.5–8.1, *p* < 0.001). In addition, older patients were again less likely to undergo a RRCM than younger patients; but only significantly so if they had a predictive test (HR 0.96, 95 % CI 0.94–0.98, 0 < 0.001).

For *diagnostically* tested patients, the mean time between DNA test results and delayed RRCM decreased over time from 108.8 months (SD 5.1) in 1995–2000 to 67.3 months in 2001–2009 (SD 5.9, *p* < 0.001) (Fig. 1d). There was an increase in the percentage of patients who had a RRCM 5 years after DNA test result from 17.5 to 37.5 %.

## Discussion

Our data show that, in the time period 2001–2009, *BRCA1/2* mutation carriers who were not aware of their carrier status at the time of their breast cancer diagnosis, had diagnostic DNA testing sooner after diagnosis than those in the time period 1995–2000. Additionally, the proportion of breast cancer patients who had predictive DNA testing increased significantly over time. This shift towards more predictive testing probably reflects the greater availability, completeness and acceptance of DNA testing over time, and a decrease in the time required to report test results. However, a survival bias cannot be ruled out, as patients must have survived long enough to be recruited in the HEBON study. Importantly, our data also clearly indicate that women known to be carrier before breast cancer diagnosis opted significantly more often for an immediate RRCM than those who had a DNA test after cancer diagnosis. Apparently, for the decision to undergo an immediate RRCM, DNA test results are more important to both patients and treating specialists than being at risk of having hereditary breast cancer only. This is relevant, since there are concerns that RGCT will increase the percentage of women opting for an immediate RRCM not only in carriers, but also in women without a pathogenic mutation. However, this finding suggests that it is unlikely for RGCT to make women without a mutation opt more often for an immediate RRCM. There was, however, no significant increase over time in the frequency of RRCM in patients who had a predictive test. Similarly, no trend over time was seen for the uptake of delayed RRCM in both patients who had a predictive test and those who had a diagnostic test. Patients who had a diagnostic test opted for RRCM sooner after breast cancer diagnosis in the more recent time period.

Overall, 34 % of the patients who developed unilateral breast cancer after a predictive DNA test had an *immediate* RRCM. This is only slightly less than the percentage reported by Cortesi et al.[19], who observed that 42 % of women who became aware of their carrier status within 1 month after breast cancer diagnosis opted for a RRCM, although it is not clear whether this was performed at the time of primary surgery or thereafter. As to our knowledge, no other studies provide explicit information on the timing of DNA testing (i.e., predictive or diagnostic), which makes it difficult to compare results. However, two American studies provide some information. King et al. [26] observed that 54 % of affected carriers had a RRCM within 1 year after diagnosis. Chung et al. observed that 13/16 (81 %) of women diagnosed with breast cancer between 1995 and 2008 who were *BRCA1/2* mutation carriers, had an immediate RRCM. Contrary to our findings, they observed an increase in the frequency of immediate RRCM over time, in both mutation carriers and in women without (knowledge of) a *BRCA1/2* mutation [27]. The increase in RRCMs over time appears to be more common in the United States, and particularly in women without an increased risk of contralateral breast cancer [26, 28–30].

In line with data from an earlier study [12], 48 % of affected carriers in our study had a *delayed* RRCM. Interestingly, patients who had a diagnostic DNA test and a delayed RRCM tended to opt for this surgery sooner after breast cancer diagnosis in the more recent time period (from a median of 77–27 months). This may be explained, at least in part, by the fact that the time between breast cancer diagnosis and DNA test results decreased in this group from 28 months in patients diagnosed 1995–2000 to 14 months in patients diagnosed 2001–2009. It is unclear whether this reflects primarily an earlier decision to undergo RRCM, reduced waiting times for surgery or differences in advice from the multidisciplinary team.

Our study had several limitations that should be noted. Women treated for breast cancer in 1995–2000 differed from those treated in 2001–2009 in age at diagnosis, follow-up time, and possibly also were subject to survival bias. Second, our sample with data available on risk reducing mastectomy was rather small. Third, although there may have been between-hospital variation in the criteria used for performing a RRCM (e.g., disease-free time since diagnosis or nodal status), use of MRI at breast cancer diagnosis, or availability and quality of breast reconstruction, we did not have data to address this question. Finally, we analyzed *BRCA1* and *BRCA2* mutation carriers together, also in view of the small numbers of *BRCA2* mutation carriers. However, we do not expect significant differences between *BRCA1* and *BRCA2* mutation carriers in choice of (risk reducing) breast surgery, since both groups probably received similar information about the risk of (contralateral) breast cancer.

Our study also has a number of noteworthy strengths. First, unlike most earlier studies, we have reported results on the choice of RRCM separately for mutation carriers who developed breast cancer following a predictive DNA test, and those mutation carriers who had a diagnostic DNA test following their breast cancer diagnosis. Second, we investigated trends over time; something that was not done in some earlier, large studies of risk reducing surgery in *BRCA1/2* mutation carriers with breast cancer [10, 11, 31].

### Implications for daily practice

Our results indicate that knowledge of one’s carrier status at the time of breast cancer diagnosis is important in decisions about risk reducing mastectomy, and that for those without this knowledge, especially in young breast cancer patients, there may be a need for RGCT to guide treatment decisions.

Since the use of neo-adjuvant chemotherapy is increasing, it is expected that an increasing number of patients will be able to receive DNA test results before primary surgery and incorporate these in their treatment decisions. With such information at hand, both breast cancer specialists and their high-risk breast cancer patients will hopefully be able to make more informed decisions about the most appropriate, individualized treatment.

## Electronic supplementary material

Below is the link to the electronic supplementary material.

Supplementary material 1 (DOCX 30 kb)

Supplementary material 2 (DOCX 214 kb)
