# Supplementary material for: Timing of risk reducing mastectomy in breast cancer patients carrying a BRCA1/2 mutation: retrospective data from the Dutch HEBON study
Source: Fam Cancer. 2015 Feb 21;14(3):355–63. doi: 10.1007/s10689-015-9788-x (PMC4559099; doi:10.1007/s10689-015-9788-x)
Supplement: Supplementary file 1 — Supplementary material 1 (DOCX 30 kb) [file 10689_2015_9788_MOESM1_ESM.docx]

**Supplementary figure 1**

Title: Possibilities of timing of DNA testing and risk reducing surgery

Legend: timeline with possible timing of DNA testing and risk reducing surgery in relation to breast cancer diagnosis and surgery

^Note: in our database risk reducing surgery never took place before a predictive DNA test; moreover, in the analyses we only considered patients with a risk reducing contralateral surgery after a first primary breast cancer diagnosis.^
