# Supplementary material for: Timing of risk reducing mastectomy in breast cancer patients carrying a BRCA1/2 mutation: retrospective data from the Dutch HEBON study
Source: Fam Cancer. 2015 Feb 21;14(3):355–63. doi: 10.1007/s10689-015-9788-x (PMC4559099; doi:10.1007/s10689-015-9788-x)
Supplement: Supplementary file 2 — Supplementary material 2 (DOCX 214 kb) [file 10689_2015_9788_MOESM2_ESM.docx]

**Supplementary figure 2**

Title: Types of primary surgery

Legend: Surgical treatment of first breast cancer diagnosis (BCT: Breast Conserving Therapy; ULM: Unilateral Mastectomy; BLM: Bilateral Mastectomy).
